# The role of experience in children’s discrimination of unfamiliar languages

**DOI:** 10.3389/fpsyg.2015.01587

**Published:** 2015-10-15

**Authors:** Christine E. Potter, Jenny R. Saffran

**Affiliations:** Department of Psychology, University of Wisconsin–Madison, MadisonWI, USA

**Keywords:** speech perception, language discrimination, second language learning, bilingualism, attention

## Abstract

Five- and six-year-old children (*n* = 160) participated in three studies designed to explore language discrimination. After an initial exposure period (during which children heard either an unfamiliar language, a familiar language, or music), children performed an ABX discrimination task involving two unfamiliar languages that were either similar (Spanish vs. Italian) or different (Spanish vs. Mandarin). On each trial, participants heard two sentences spoken by two individuals, each spoken in an unfamiliar language. The pair was followed by a third sentence spoken in one of the two languages. Participants were asked to judge whether the third sentence was spoken by the first speaker or the second speaker. Across studies, both the difficulty of the discrimination contrast and the relation between exposure and test materials affected children’s performance. In particular, language discrimination performance was facilitated by an initial exposure to a different unfamiliar language, suggesting that experience can help tune children’s attention to the relevant features of novel languages.

## Introduction

In multilingual environments, children must determine which individuals speak the same language, and which individuals speak different languages. While some children regularly hear multiple dialects or languages, others may have very little experience hearing different-sounding languages. When multiple unfamiliar languages are present, can young children differentiate between them and appropriately link different speakers to different languages? Does exposure to a new language change children’s ability to differentiate among other unfamiliar languages?

At birth, infants are sensitive to some key differences between languages. Newborns can discriminate their native language from a non-native language (Mehler et al., 1988; Moon et al., 1993) and can also discriminate between two unfamiliar languages if they are sufficiently distinct (Mehler and Christophe, 1994, 1995; Nazzi et al., 1998). Rhythmic properties are particularly useful for language discrimination in infancy. For example, English and Dutch are considered to be stress-timed, meaning that stressed and unstressed syllables have different durations. In contrast, syllable duration is less variable in syllable-timed languages such as French and Italian, and in Japanese, the rhythmic unit is the mora (Otake et al., 1993). Using these cues, newborn French infants can discriminate between English and Japanese, which have distinctive rhythmic properties, but do not distinguish between English and Dutch, which belong to the same non-native rhythmic class (Nazzi et al., 1998).

As they gain experience with their native language, infants learn to attend primarily to features that are relevant for that language, (e.g., Werker and Tees, 1984; Kuhl et al., 2006), which may in turn change their perception of other languages. After even just a few months of life, infants no longer make distinctions they would have made as newborns, failing to discriminate between unfamiliar languages unless one is similar to their own. For example, English-learning 2-month-olds can discriminate their native English from unfamiliar Japanese or Italian (Mehler et al., 1988; Christophe and Morton, 1998), but fail to differentiate between Japanese and French despite their rhythmic differences (Christophe and Morton, 1998). They are more successful when one of the languages is more similar to a familiar language: English-learning infants can discriminate Dutch from Japanese, demonstrating that experience with one language (English) may influence the perception of a novel but similar-sounding language (Dutch). Further emphasizing the difference in how familiar and unfamiliar languages are perceived, 5-month-old American infants successfully discriminate their own language from other languages within the same rhythmic class (e.g., English vs. Dutch), but not two unfamiliar languages from the same class (e.g., German vs. Dutch, or Spanish vs. Italian; Nazzi et al., 2000). Interestingly, in contrast with the 2-month-olds studied by Christophe and Morton (1998), these slightly older 5-month-olds could also discriminate between two unfamiliar languages when they came from distinct rhythmic classes (e.g., Italian and Japanese). Rhythm, therefore, may remain an important cue, but it is not the only property to which infants attend, at least when they hear a familiar language. Thus, as infants get older, there appear to be two factors that contribute to their ability to discriminate between unfamiliar languages: increased experience with their native language, and the specific properties of the languages to be discriminated.

Beyond infancy, children remain sensitive to the differences between familiar and unfamiliar languages. Five-year-old children use the language spoken by an unknown individual to guide social preferences, demonstrating that they consider differences between their native language and unfamiliar languages to be meaningful (Kinzler et al., 2007). Likewise, young children are more likely to imitate an actor who speaks their language than a foreign speaker (Buttelmann et al., 2013; Howard et al., 2015). Preschool aged children are also sensitive to more subtle distinctions, preferring speakers with their native accent to those with an unfamiliar accent even within their native language (Kinzler et al., 2007, 2011; Wagner et al., 2014b).

When children are confronted with only unfamiliar accents or dialects, however, the differences between languages become more difficult to detect (Floccia et al., 2009; Wagner et al., 2014a). In a study by Stockmal et al. (1994), 4- and 5-year-old children heard pairs of utterances in a variety of non-native languages and were asked to judge whether the two tokens were drawn from the same or different languages. Children struggled with this task and were unable to determine whether two utterances came from the same language unless they were provided with additional cues, such as hearing the identical phrase for both utterances, or hearing both tokens spoken by the same talker. When languages are unfamiliar, young listeners may have trouble determining which features to prioritize, making it difficult for them to recognize when languages are the same or different.

Like children, adults’ ability to discriminate between languages is affected by both the familiarity of a language and its acoustic properties. Unsurprisingly, adults are most accurate at identifying their native language (Muthusamy et al., 1994). Nevertheless, adults can describe some features of unfamiliar languages and can discriminate between two unknown languages at above-chance levels, though there is wide individual variability (Lorch and Meara, 1989, 1995). Listeners report using prosody, pitch, and voice characteristics to make judgments, and like infants, have greater difficulty discriminating between languages within the same rhythmic class (Muthusamy et al., 1994; Stockmal et al., 2000).

Even brief exposure can influence adults’ ability to recognize a new language. Adults who watched short cartoons in Japanese were subsequently better able to discriminate Japanese from other unknown languages (Bond et al., 1998). While all participants were relatively successful in identifying Japanese when it was contrasted with typologically distinct languages (Russian and Arabic), exposure significantly decreased participants’ tendency to confuse Japanese with more similar languages (Chinese and Indonesian). As with infants, both experience with a specific language and features of that language appear to influence adults’ ability to distinguish that language from others.

Language experience also influences the perception of new languages more indirectly. Bilingual infants, who must learn the relevant contrasts for two language systems, show a different trajectory in the organization of their perceptual system than monolinguals (Bosch and Sebastián-Gallés, 1997, 2003; Byers-Heinlein and Fennell, 2014). Bilinguals retain the ability to perceive non-native contrasts longer than monolinguals, suggesting that exposure to multiple languages alters the features to which infants attend (e.g., Petitto et al., 2012). Thus, the increased variability in bilingual infants’ early environment influences the cues they consider relevant when encountering unfamiliar languages.

This differential sensitivity appears to persist later in life. Bilingual adults are better able to identify new languages than monolinguals, and knowing more than two languages further improves performance (Muthusamy et al., 1994). Moreover, later experience with multiple languages can also be advantageous; second language learners are more successful in identifying an unfamiliar language than adults who know only one language (Marks et al., 2003). Together, these studies illustrate that increased experience with multiple languages influences listeners’ sensitivities to unfamiliar languages.

The literature thus suggests that regular exposure to multiple languages changes both infants’ and adults’ perception of new languages, and for adults, even brief exposure can improve their ability to distinguish a novel target language from other unfamiliar languages. It also reveals that regular experience with multiple languages can change listeners’ perceptions of unfamiliar languages, though it is not clear how much exposure is needed. However, the factors influencing children’s language discrimination are not yet well understood, and it is not known if brief experience with a language is enough to change children’s perception of unfamiliar languages. Previous research has found that preschool-aged children do use language and accent information to make inferences about a speaker but has also suggested that they may have difficulty recognizing unfamiliar languages (e.g., Stockmal et al., 1994; Kinzler et al., 2007, 2011; Floccia et al., 2009; Wagner et al., 2014a,b). Therefore, given that adults’ ability to identify unknown languages can be improved by even short amounts of experience with that language, does brief exposure to a new language also change children’s perception of that language? Does that exposure also change their perception of other unfamiliar languages?

In the current studies, we explored children’s ability to discriminate between unfamiliar languages. In particular, we focused on the role of exposure: does experience with a novel language tune children’s attention to differences among other unfamiliar languages? In a series of studies, we briefly exposed children to unfamiliar languages; the exposure phase was then followed by a language discrimination task. Across experiments, we manipulated the difficulty of the discrimination (as a function of the similarity of the languages involved) and the relation between the materials heard during the exposure and test phases of the experiment. Our goal was to determine whether exposure to a new language would influence children’s ability to distinguish between unknown languages.

## Experiment 1

In our first study, we asked whether exposure to an unfamiliar language impacts subsequent language discrimination. First, we briefly exposed monolingual English-speaking 5-and 6-year-old children to either Mandarin (Experimental condition) or to music (Control condition). We then gave children a language discrimination task, in which we manipulated the difficulty of the discrimination (between subjects). In the Easy Contrast test condition, the two languages were typologically distinct (Spanish vs. Mandarin). In the Difficult Contrast test condition, the two languages were typologically similar (Spanish vs. Italian). The question of interest was whether the Mandarin exposure would affect subsequent language discrimination relative to the control group (who heard music during exposure).

We had three primary predictions. Given that phonological similarity affects language discrimination (e.g., Mehler et al., 1988; Nazzi et al., 2000; Stockmal et al., 2000), we predicted that children tested on the Easy contrast (Spanish vs. Mandarin) would outperform children tested on the Difficult contrast (Spanish vs. Italian). Second, we predicted that exposure to Mandarin would facilitate children’s performance on the Easy contrast; the Easy contrast included Mandarin materials, and previous work suggests that brief exposure to a target language subsequently helps adults to identify that language (Bond et al., 1998). We therefore expected that exposure to a new language would make it easier for children to discriminate that language from another unfamiliar language. Finally, we predicted that exposure to Mandarin would impair performance on the Difficult contrast by highlighting features of Mandarin not reflected in the discrimination task (Spanish vs. Italian). The features that characterize Mandarin are very different from those that distinguish Spanish and Italian. After only a few months of experience with their native language, infants no longer distinguish between two foreign languages that they would have discriminated at birth, demonstrating that exposure to one language can lead listeners to become less sensitive to features that differentiate other languages (Christophe and Morton, 1998). Therefore, we expected children in the Experimental condition to have more difficulty differentiating between similar-sounding Spanish and Italian after they heard Mandarin, as compared to children who were exposed to music in the Control condition.

### Method

We developed a child-friendly ABX task to measure language discrimination, appropriate for 5- and 6-year-olds. Pilot testing revealed that younger children struggled with this particular task. Children were briefly exposed to either Mandarin (experimental condition) or music (control condition). They were then tested in the ABX task on either Easy Contrast trials (Spanish vs. Mandarin) or Difficult Contrast trials (Spanish vs. Italian), creating a 2 × 2 (Exposure Condition by Test contrast) between-subjects design. On each ABX test trial, children heard sentences produced by speakers of two different languages (paired with still images of two cartoon monsters). They then heard a third sentence, spoken in one of the two languages. The dependent variable was the accuracy with which children identified which of the two monsters had spoken the third sentence. In the Easy Contrast condition, each test trial included a Spanish sentence paired with a Mandarin sentence. In the Difficult Contrast condition, each test trial included a Spanish sentence paired with an Italian sentence. Each participant heard only one type of exposure condition (Mandarin or music) and one type of test contrast (Easy or Difficult).

#### Participants

Participants included 80 monolingual 5- and 6-year-old children (mean age = 70 months, range: 60–83), recruited and tested at a children’s museum in the Midwestern U. S. An additional nine participants were tested and excluded because of inattentiveness (*n* = 1), or because they failed the English-only training phase (*n* = 8). Children were pseudo-randomly assigned to one of the four conditions such that in each of the four conditions, there were the same number of total participants (20) and female participants (12), and there was no difference in age between groups [*F*(3,76) = 0.14, *p* = 0.94]. The parents of all participants in Experiment 1 and the subsequent experiments provided informed consent. All experimental protocols, including procedures for obtaining informed consent, were approved by the University of Wisconsin–Madison IRB.

#### Apparatus

Children were tested individually in a quiet room, away from the museum exhibits. They sat at a table and wore headphones while looking at a 10″ × 16″ computer monitor. Stimuli were controlled by the primary experimenter, and parents sat nearby but did not interact with the child during testing. A second experimenter, who was blind to condition, coded children’s responses.

#### Materials

##### Visual stimuli

**FIGURE 1:**
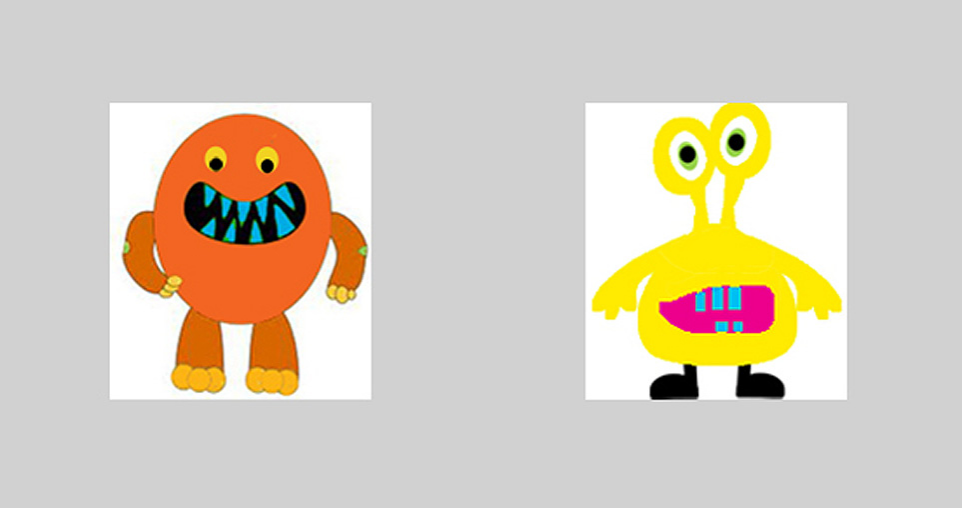
**Sample visual stimuli**.

Children viewed images of brightly colored cartoon monsters designed to be engaging for children (adapted from Amato and MacDonald, 2010). Two monsters were used in the initial English-only training phase, and four additional monsters were used in the test phase. All six monsters were easy to distinguish from one another. Images were edited to be roughly the same size and were presented in pairs against a gray background (see **Figure 1**).

##### Auditory stimuli

*Exposure*. There were two sets of auditory exposure materials, Mandarin and music. The Mandarin exposure (Experimental condition) consisted of short passages of child-directed speech, spoken by a female native Mandarin speaker. The music exposure (Control condition) was a recording of a Mozart piano sonata, matched in duration to the Mandarin passages (24 s). There were two exposure passages of each type (See Appendix for details).

*Test stimuli*. The test stimuli consisted of sentences spoken in Spanish, Italian, and Mandarin. In the Easy Contrast condition, Spanish sentences were paired with Mandarin. In the Difficult Contrast condition, the same Spanish sentences were paired with sentences from a much more similar language, Italian. All test stimuli were produced by female native speakers. There were two speakers for each test language, and each speaker produced 12 sentences to be used in testing. All sentences were matched in volume and chosen to be roughly the same duration but were otherwise left to be as natural as possible to preserve ecological validity. Different speakers produced the exposure and test sentences.

##### Other measures

Parents also filled out brief surveys about their child’s exposure to languages other than English. Any participant who reported significant exposure to another language (≥1 h a day) was excluded from all analyses.

#### Procedure

##### Training phase

Children were introduced to the task by being told that they were going to play a game with talking monsters. The training phase used a male and female speaker in order to make it as easy as possible for children to understand the task. They were asked to look at the screen where the two practice monsters appeared and were told that the first monster was going to talk. The experimenter pointed to the monster on the left side and that monster was illuminated, while the other monster was dulled, and the child heard a sentence from a female speaker. Next, the participant was told that the other monster would speak. This time, the experimenter pointed the monster on the right, which was illuminated, while the monster on the left was dulled, and the child heard a male speaker. The experimenter then told the child that one of the two monsters was going to say something else and that it was her job to guess which monster it was. The child heard a third sentence, produced by the male speaker, and was asked to indicate which monster had spoken, with both monsters equally illuminated. She could either point to the picture or give a verbal response (e.g., *the yellow one*). Children were given feedback, and those who made errors were given an opportunity to hear the trial again and then were corrected if they still chose the wrong monster. If children responded incorrectly a second time, the experimenter corrected them and had them point to the correct monster before going on to the next trial.

There were a total of four English practice trials. The monster on the left always spoke first, followed by the monster on the right. The experimenter always told the child which monster was going to speak before it was illuminated, and then cued the child to respond after the test sentence. The same male and female speakers were used throughout, and each speaker was consistently paired with a single monster (e.g., the yellow monster was always the male speaker). The target was equally likely to be the male or female speaker, and order was counterbalanced such that the monsters appeared equally often on both sides and the target was equally likely to be the first or second speaker. Children continued to receive feedback throughout training. Those children who could not correctly answer at least two trials without assistance, or who used a consistent strategy (e.g., always picking the second monster) were considered to have failed the practice trials.

##### Exposure phase

Immediately following the training phase, children were told they were going to watch a short movie. They then listened to either the Mandarin (Experimental condition) or music (Control condition) exposure materials while viewing videos of nature scenes, intended to maintain the children’s interest. This phase lasted approximately 25s.

##### Test phase

Immediately following the exposure phase, children were told that they were going to meet new monsters that would talk to them. The procedure was the same ABX task used in training, but children received only neutral feedback after their responses. Each of the four monsters used in testing was consistently paired with a particular speaker. In the Easy Contrast condition, two monsters spoke Spanish and two spoke Mandarin. In the Difficult Contrast condition, two monsters spoke Spanish and two spoke Italian. On each trial, the child heard one speaker of Spanish and one speaker of the contrast language (Mandarin or Italian, depending on condition). The target sentence was produced by the same speaker who had produced one of the first two sentences. Children were tested in two blocks of four trials each. After the first four test trials, children in the Experimental condition heard a second exposure passage of Mandarin, while children in the Control condition heard a second musical passage. They then completed four additional test trials. The entire study lasted about 5 min.

### Results

**FIGURE 2:**
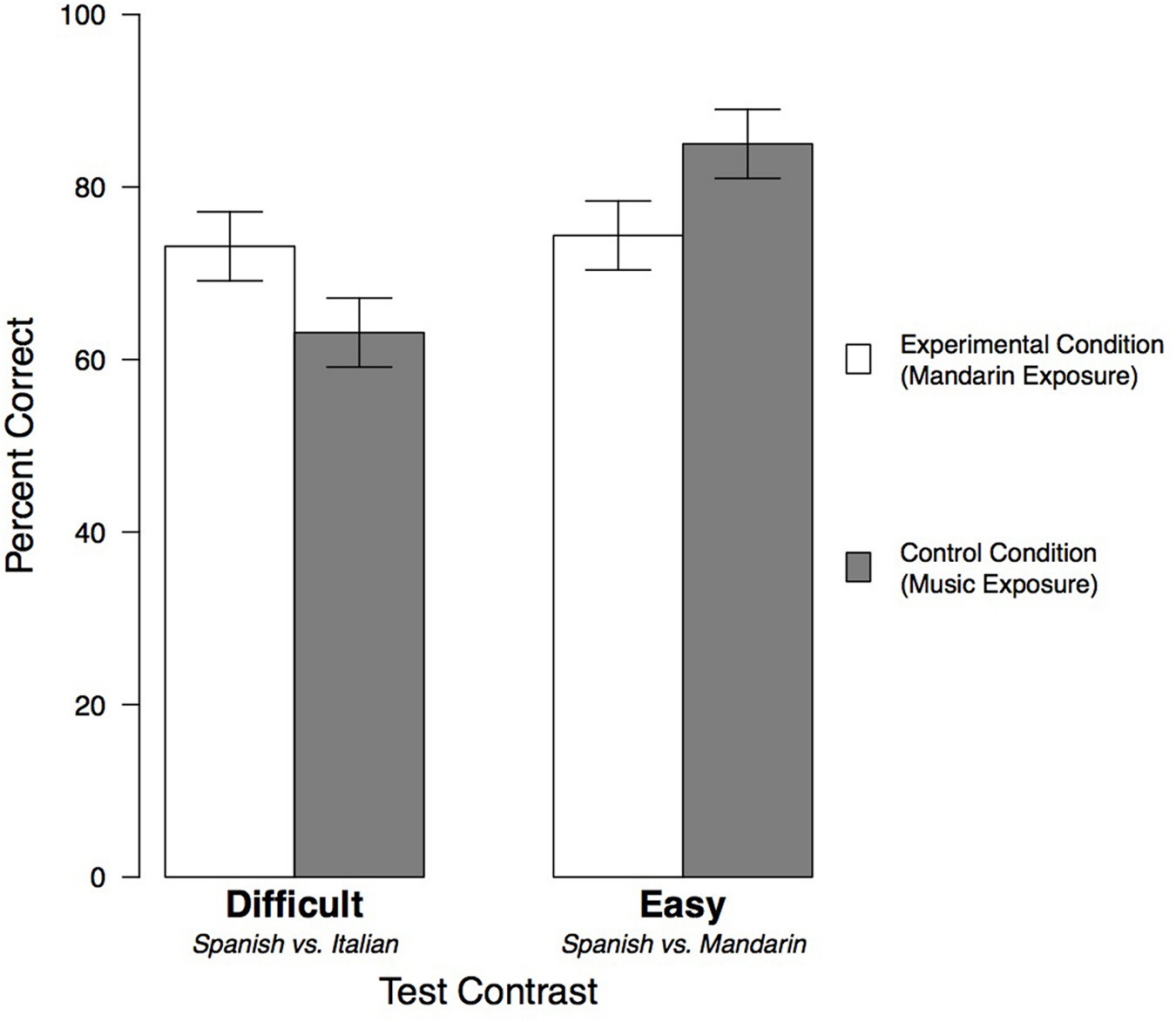
**Children’s performance in Experiment 1, divided by Test contrast and Exposure Condition.** Error bars indicate standard errors.

The means and standard errors for each condition are shown in **Figure 2** and **Table 1**. To explore the effects of Exposure Condition (Mandarin vs. Music) and Test contrast (Difficult vs. Easy) on children’s ability to perform the language discrimination task, we performed a 2 × 2 ANOVA. There was a significant interaction [*F*(1,76) = 6.47, *p* = 0.01, $\eta_{p}^{2}$ = 0.080], so our analysis used Type III Sum of Squares. After controlling for the interaction, the main effects were not significant [Exposure Condition: *F*(1,76) = 3.13, *p* = 0.08; Test: *F*(1,76) = 0.049, *p* = 0.83]. These results suggest that our manipulations did affect children’s discrimination, so we conducted a series of follow-up tests to test our hypotheses.

**Table 1:** Mean percent correct and standard deviations, across all studies.

	Study 1		Study 2	Study 3
Exposure type	Music	Mandarin	Italian	English
**Test contrast**				
Difficult (Spanish vs. Italian)	63.1% (17.9)	73.1% (17.8)	67.5% (19.6)	58.8% (13.5)
Easy (Spanish vs. Mandarin)	85.0% (18.0)	74.4% (17.9)	86.3% (15.1)	76.3% (20.2)

*Note that for the Difficult Test Contrast, Mandarin is the Incongruent Exposure and Italian is the Congruent Exposure, while for the Easy Test Contrast, Mandarin is the Congruent Exposure, and Italian is the Incongruent Exposure.*

Our first hypothesis, namely that children would perform better on the Easy contrast (Spanish vs. Mandarin) than the Difficult contrast (Spanish vs. Italian), was not supported by the overall ANOVA, as the main effect of Test was not significant. However, because the interaction revealed significant differences, we conducted a series of pairwise comparisons, using Tukey’s HSD to control for multiple comparisons. The first contrast revealed differences for children in the Control group (exposed to music). For these participants, children tested on the Easy contrast outperformed those tested on the Difficult contrast (*p* = 0.001, Cohen’s *d* = 1.25). Thus, in the absence of exposure to an unfamiliar language, children found it easier to discriminate between typologically distinct languages (Spanish and Mandarin; Easy contrast) than typologically similar languages (Spanish and Italian; Difficult contrast), partially supporting our initial prediction.

Our second hypothesis was that exposure to Mandarin would lead to improved performance on the Easy contrast (Spanish vs. Mandarin). To test this hypothesis, we used our second pairwise comparison, which compared performance on the Easy contrast for children who were exposed to Mandarin (Experimental condition) versus children who were exposed to music (Control condition). Contrary to our prediction, children exposed to Mandarin performed numerically worse on the Easy contrast than children exposed to music (74.4% vs. 85.0% correct), although this difference was not significant (*p* = 0.25).

Third, we expected Mandarin exposure to impair children’s performance on the Difficult contrast (Spanish vs. Italian). However, when our final contrast compared the Control and Experimental groups tested on the Difficult contrast, the results were again in the opposite direction of our prediction: Children performed numerically better after exposure to Mandarin than after exposure to music (73.1% vs. 63.1% correct), though this difference was not significant (*p* = 0.30).

While these individual comparisons were not significant, the significant interaction in the main ANOVA nevertheless suggests that exposure to Mandarin differentially affected children’s ability to make Easy and Difficult discriminations. Contrary to our predictions, the difference between the Easy and Difficult Test contrasts was no longer present following exposure to Mandarin. Thus, the Mandarin exposure condition appears to have led to *better* performance on the Difficult contrast and *worse* performance on the Easy contrast, relative to the Control groups.

Finally, we examined potential effects of gender, age, and block. There were no effects of gender or block in this study or any of the subsequent studies, so we did not include those factors in our models. However, when we used a general linear model that included age (in months) as a continuous variable, we saw a marginal effect of age [*F*(1,78) = 3.175, *p* = 0.08]. Older children performed slightly better on the task, but there were no interactions between age and either of our experimental variables (Exposure type and Test contrast), suggesting that older and younger children showed similar patterns of performance.

### Discussion

Experiment 1 yielded mixed support for our initial predictions. While children did not perform differently on the two types of test contrast overall, children in the Control condition (music exposure) performed better on the Easy contrast than the Difficult contrast. In other words, without any additional language exposure, children found it easier to differentiate between Spanish vs. Mandarin than to distinguish between Spanish vs. Italian. This result is consistent with the prior literature, and suggests that in the absence of experience with a new language, children do find it easier to discriminate between languages that sound different than languages that sound similar.

However, contrary to our other two predictions, exposure to Mandarin appeared to *hinder* performance on the Easy contrast (Spanish vs. Mandarin) and *facilitate* performance on the Difficult contrast (Spanish vs. Italian), relative to the Control group. Unlike children in the Control condition, children who were exposed to Mandarin no longer performed better on the Easy contrast than on the Difficult contrast, suggesting that exposure to Mandarin may have a different effect on the two types of Test contrasts. It could be that this difference was due to the relative difficulty of the ABX task; perhaps exposure to a new language is particularly advantageous for listeners who are subsequently faced with a difficult language discrimination. Another possibility is that the overlapping use of Mandarin during both the exposure and test phases affected the pattern of results. That is, hearing the same language during the two phases of the experiment may have made the discrimination task more challenging. Given that Mandarin was the only language used in exposure in Experiment 1, it is not possible to adjudicate between these explanations. Therefore, our second study was designed to examine the relative contributions of the difficulty of the contrast and the relation between the exposure and test materials on children’s ability to discriminate between languages.

## Experiment 2

In our first experiment, we saw that exposure to Mandarin had different effects on children’s ability to make Easy and Difficult discriminations. However, from the existing data, we cannot determine if that pattern was due to the repeated use of a single language in two phases of the experiment, or if that pattern was unique to Mandarin. For our second study, we manipulated the language that participants heard during the exposure phase. Children heard an exposure language that was either *Congruent* or *Incongruent* with the test materials. In Experiment 1, for children tested on the Easy Test contrast (Spanish vs. Mandarin), Mandarin exposure can be considered a *Congruent Language* with respect to the test materials, since children heard Mandarin in both phases. In contrast, for children tested on the Difficult contrast (Spanish vs. Italian), Mandarin can be considered an *Incongruent Language*, as there was no relation between the exposure and test stimuli. **Table 2** illustrates this relation and highlights the terminology being used. Note that given the structure of Experiment 1, the congruence of the exposure phase was confounded with the difficulty of the test contrast.

**Table 2:** Congruency of exposure language and test contrast.

	Exposure language		
Test contrast	Mandarin Study 1	Italian Study 2	English Study 3
Difficult (Spanish vs. Italian)	Incongruent Language	Congruent Language	Incongruent-Familiar
Easy (Spanish vs. Mandarin)	Congruent Language	Incongruent Language	Incongruent-Familiar

Therefore, Experiment 2 was designed to separate the potential effect of hearing a congruent language from the difficulty of the contrast. To do so, we added a new exposure language: Italian. For children tested on the Difficult contrast (Spanish vs. Italian), the Italian exposure is *Congruent –* the exposure language also participates in the test contrast. Conversely, for children tested on the Easy contrast (Spanish vs. Mandarin), Italian exposure is *Incongruent –* the exposure language does not participate in the test contrast. We can then compare children tested in Experiment 2 to children exposed to Mandarin in Experiment 1. Each language can then act as its own control, allowing us to determine whether the difficulty of the contrast (Easy vs. Difficulty) or the relation between exposure and test materials (Congruent Language vs. Incongruent Language) impacts children’s ability to discriminate between languages. If the congruency between exposure and test materials affects performance, then we would expect children tested on both Test contrasts to perform better after exposure to an Incongruent Language. On the other hand, if the difficulty of the Test contrast affects performance, then we would expect children to perform equally well after exposure to either a Congruent or Incongruent Language.

We had two predictions for this second experiment. The first was that we expected children to perform better overall on the Easy contrast than the Difficult contrast. We also predicted that the congruence of the exposure and test materials would affect children’s discrimination performance: children tested on both contrasts would perform better after exposure to an Incongruent Language than after exposure to a Congruent Language. Specifically, based on the results of Experiment 1, we hypothesized that children tested on the Easy contrast would perform better after exposure to Italian (Incongruent Language) than Mandarin (Congruent Language), while children tested on the Difficult contrast would perform better after exposure to Mandarin (Incongruent Language) than Italian (Congruent Language). We did not include data from children in the Control condition in Experiment 1 because music is neither congruent nor incongruent with respect to these test materials, and therefore cannot provide insight into the role played by congruency.

### Method

The procedure was identical to Experiment 1, except that all children heard Italian during the exposure phase. They were tested on the same Easy and Difficult test contrasts as in Experiment 1 (between-subjects).

#### Participants

Participants were a new sample of 40 children (24 female), recruited from the same population as Experiment 1 (mean age = 70 months, range: 60–82). Five additional participants were excluded due to hearing loss (*n* = 1), inattentiveness (*n* = 1), or failing the practice trials (*n* = 3). Children were assigned to conditions to match the sample size, age, and gender makeup of Experiment 1. The four conditions were Congruent-Easy (Experiment 1), Congruent-Difficult (Experiment 2), Incongruent-Easy (Experiment 2), and Incongruent-Difficult (Experiment 1). There was no difference in age between participants in Experiment 2 and participants in the experimental groups from Experiment 1 [*F*(3,76) = 0.10, *p* = 0.96].

#### Materials

All materials were identical to those used in Experiment 1, except that instead of music or Mandarin materials, the exposure stimuli were in Italian (See Appendix for details). The new exposure stimuli consisted of naturally produced, child-directed speech, spoken by a female native Italian speaker who did not produce any of the test materials.

### Results

As in Experiment 1, we began by performing a 2 × 2 ANOVA to examine the effects of Exposure Language and Test contrast on children’s ability to discriminate between languages. We coded Exposure Language as either a Congruent Language (e.g., children from Experiment 1 who were exposed to Mandarin and then tested on the Easy contrast: Spanish vs. Mandarin) or Incongruent Language (e.g., children from Experiment 2 who were exposed to Italian and then tested on the Easy contrast).

**FIGURE 3:**
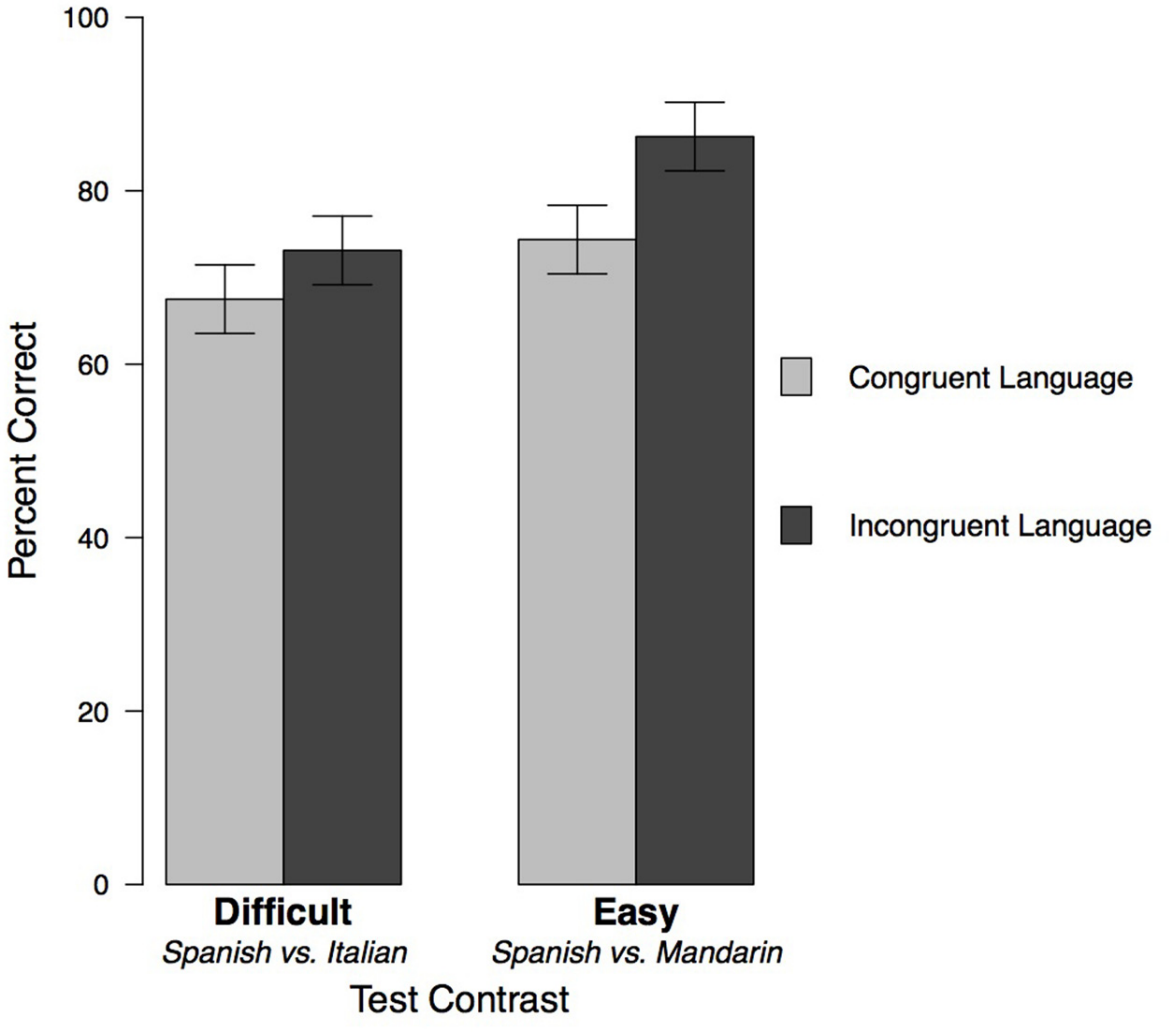
**Children’s performance on the discrimination task in Experiment 1 (Mandarin exposure) and Experiment 2 (Italian exposure), divided by the relation between Exposure Language and test materials.** Error bars indicate standard errors.

In this ANOVA, the interaction was not significant [*F*(1,76) = 0.625, *p* = 0.43], so we used Type II Sum of Squares for our interactive model. We found significant main effects of both Test contrast [*F*(1,76) = 6.40, *p* = 0.01, $\eta_{p}^{2}$ = 0.076] and Exposure Language [*F*(1,76) = 4.90, *p* = 0.03, $\eta_{p}^{2}$ = 0.059]. See **Figure 3** and **Table 1** for means and standard errors.

The significant main effect of Test contrast showed that children performed better on the Easy contrast than the Difficult contrast. Consistent with our first prediction, children were better able to discriminate between more distinct languages (Spanish and Mandarin) than relatively similar languages (Spanish and Italian) after exposure to both Congruent and Incongruent Languages.

Our second hypothesis was also supported by the significant main effect of Exposure Language. Across both Test contrasts, children were better able to discriminate between unfamiliar languages after they had heard an Incongruent Language during the exposure phase, relative to a Congruent Language. That is, hearing Mandarin facilitated test performance on the contrast that did not include Mandarin (Difficult contrast: Spanish vs. Italian), while hearing Italian facilitated performance on the contrast that did not include Italian (Easy contrast: Spanish vs. Mandarin). Taken together, the data from Experiments 1 (Mandarin exposure) and 2 (Italian exposure) suggest that contrary to our initial predictions, exposure to an unfamiliar language (Incongruent Language exposure) facilitates discrimination between two additional unfamiliar languages, as compared to exposure to a language that is also used in the discrimination task (Congruent Language exposure).

We also observed a significant effect of age [*F*(1,78) = 9.85, *p* = 0.002, $\eta_{p}^{2}$ = 0.112], with older children performing better across conditions. However, when we entered age (in months) as a continuous variable into an interactive linear model, along with Exposure and Test contrast, there was a marginal interaction of Age by Test contrast [*F*(1,72) = 2.91, *p* = 0.09, $\eta_{p}^{2}$ = 0.039], but no significant effects. Therefore, we can again conclude that older and younger children showed similar patterns of performance across conditions.

### Discussion

The results from our second experiment supported our hypotheses. As expected, children were better able to make an Easy language discrimination (Spanish vs. Mandarin) than a Difficult language discrimination (Spanish vs. Italian) across exposure languages. More interestingly, we replicated our surprising finding from Experiment 1: exposure to a language not involved in the test contrast (Incongruent) facilitated children’s ability to discriminate between two other unfamiliar languages. Children’s performance on the Difficult contrast (Spanish vs. Italian) improved after exposure to Mandarin (tested in Experiment 1), while performance on the Easy contrast (Spanish vs. Mandarin) improved after children were exposed to Italian (Experiment 2). These findings support the hypothesis that the relation between the Exposure Language and Test materials impacts language discrimination in a surprising way: discrimination is actually better when the exposure language is incongruent with the test languages.

One possible explanation for this pattern of results is that a different language could help children focus on the relevant features for making discriminations, rather than drawing their attention to properties that may not distinguish two unknown languages. Marks et al. (2003) have speculated that “phonetic distance” can help highlight relevant features for listeners when they encounter new languages. They support this suggestion by noting that German speakers were more accurate than Spanish speakers in telling Indonesian from Japanese, perhaps because both Indonesian and Japanese are more rhythmically distinct from stress-timed German than syllable-timed Spanish. Thus, in our task, Mandarin might be similarly helpful in highlighting features of Spanish and Italian that are more useful than their overlapping rhythm. Likewise, hearing Italian prior to the Easy contrast (Spanish vs. Mandarin) could draw attention to differences that can exist between languages.

However, it is unclear if distance alone explains why the Incongruent Language facilitates performance. We have suggested that the relation between exposure and test materials changes the properties to which children attend, but there could be any number of factors affecting children’s attention in this discrimination task. Another possible influence is the novelty of the unfamiliar exposure language. A recent study found that hearing a story spoken in an unfamiliar foreign accent improved preschool-aged children’s comprehension, possibly because the novelty increased their overall attention (Barker and Turner, 2014). In Experiments 1 and 2, both the Congruent and Incongruent languages were unfamiliar, making it impossible to determine if the novelty of the exposure language is also helpful in directing children’s attention.

In Experiment 3, we wanted to determine whether the novelty of the unfamiliar Incongruent Language exposure was in fact contributing to the effects seen in Experiments 1 and 2. To test this possibility, we wanted to expose children to a language that was incongruent with the test materials, but not novel. Therefore, in our third study, children heard a familiar language during the exposure phase (English), which allowed us to disentangle the potential effects of novelty and incongruence with the test materials.

## Experiment 3

Experiment 3 was designed to test the hypothesis that the unfamiliarity of the Incongruent exposure language, as well as its lack of inclusion in the test materials, helped guide children’s attention and allowed them to more successfully discriminate between languages. Using the same paradigm, we first exposed children to English, a familiar language that was unrelated to the Test materials, and compared the performance of those children who heard English during exposure to that of children in prior studies who heard either a Congruent or Incongruent Language. One possibility is that any language that is not related to test materials might facilitate children’s discrimination, regardless of whether it is familiar to the children, by highlighting the fact that languages can sound different. If that is the case, we would expect children exposed to English (familiar, but incongruent) to perform similarly to children exposed to an unfamiliar Incongruent Language. On the other hand, if the novelty of the Incongruent Language contributed to the facilitation seen in earlier experiments, then we would expect children exposed to English (Incongruent-Familiar Language) to perform worse than children exposed to Incongruent-Unfamiliar Languages (Mandarin in Experiment 1 and Italian in Experiment 2).

In our third experiment, we again expected to see children perform better on the Easy contrast than the Difficult contrast. More interestingly, we also expected the novelty of the Incongruent Language to be important and simply hearing a language unrelated to the test materials would not facilitate discrimination. We therefore predicted that children exposed to English would perform worse than children exposed to an Incongruent-Unfamiliar Language.

### Method

The procedure was identical to Experiments 1 and 2, except that children heard English during the exposure phase. They were then tested on the same Easy and Difficult test contrasts as in Experiments 1 and 2 (between-subjects).

#### Participants

Participants were 40 children (24 female), recruited from the same population as Experiments 1 and 2 (mean age = 70 months, range: 60–81). Three additional participants were tested but excluded due to failing the practice trials (*n* = 2) or not following directions (*n* = 1). There were 20 children in each new condition, and was no difference in age between participants in any of the groups in Experiments 1, 2, and 3 [*F*(7,152) = 0.25, *p* = 0.97].

#### Materials

All materials except the exposure stimuli were identical to those used in Experiment 1. The new exposure stimuli consisted of naturally produced, child-directed English speech, spoken by a female native English speaker who did not produced any of the materials used in the training phase. To minimize differences between hearing a familiar language and the foreign languages used in prior experiments, the sentences were drawn from three different story books and thus were unconnected to one another and did not create a coherent narrative (See Appendix for details).

### Results

**FIGURE 4:**
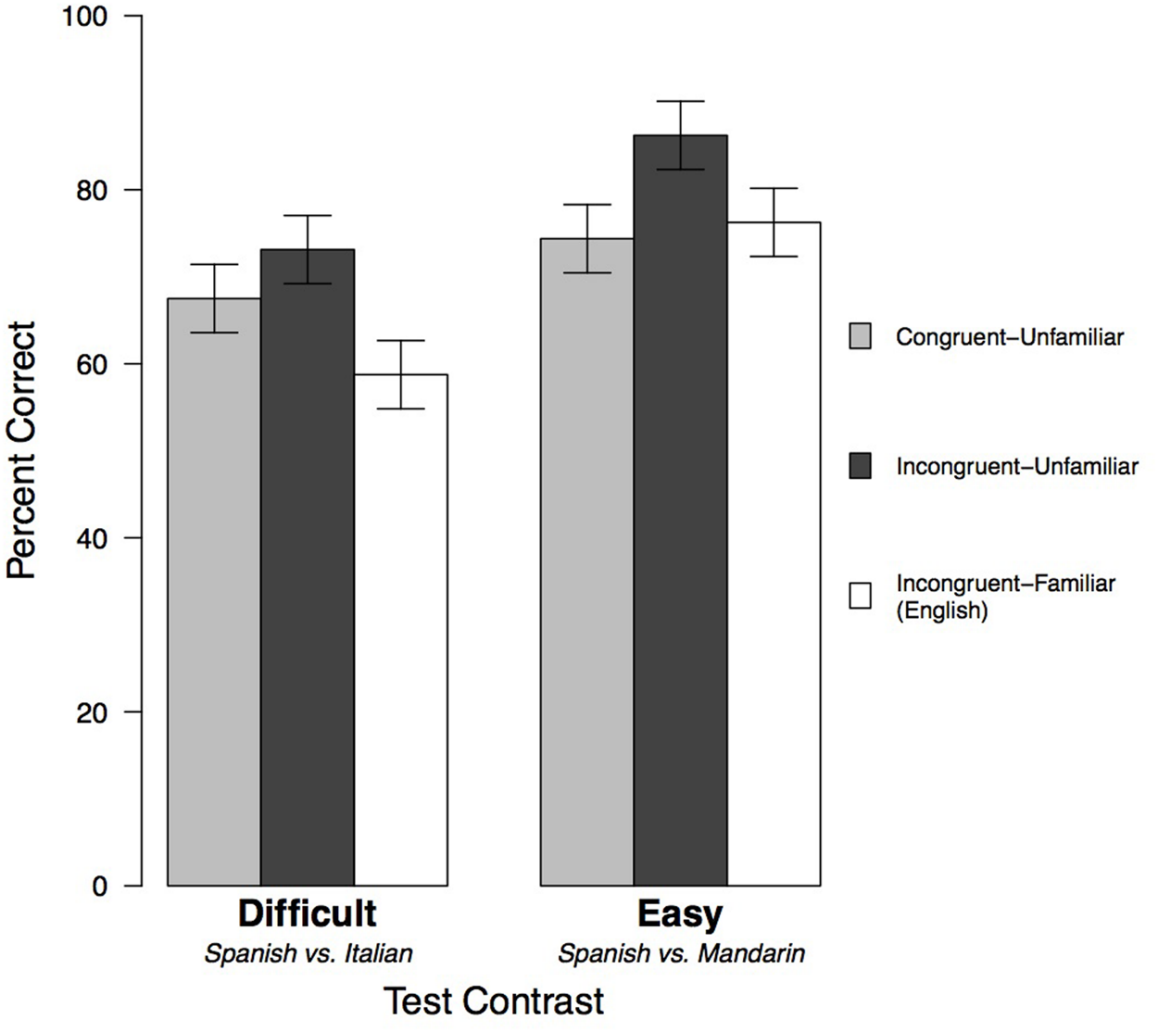
**Mean performance on the discrimination task, including data from children exposed to English in Experiment 3.** For the Difficult contrast, the Incongruent-Unfamiliar exposure language was Mandarin (Experiment 1), and the Congruent-Unfamiliar Language was Italian (Experiment 2). For the Easy Exposure contrast, the Incongruent-Unfamiliar Language was Italian (Experiment 2) and the Congruent-Unfamiliar Language was Mandarin (Experiment 1). Error bars indicate standard errors.

In Experiment 3, we compared children who were exposed to English (Incongruent-Familiar Language) to children who had been exposed to Congruent-Unfamiliar and Incongruent-Unfamiliar Languages in Experiments 1 and 2. We used a 3 × 2 ANOVA to explore the effects of Exposure Language [Congruent-Unfamiliar, Incongruent-Unfamiliar, or English (Incongruent-Familiar)] and Test contrast on children’s ability to perform the discrimination task. Our initial ANOVA showed that the interaction was not significant [*F*(2,114) = 0.93, *p* = 0.40], so we again used Type II sum of squares in our model. The model revealed significant main effects of both Exposure Language [*F*(2,114) = 5.14, *p* = 0.007, $\eta_{p}^{2}$ = 0.083] and Test contrast [*F*(1,114) = 15.26, *p* = 0.0002, $\eta_{p}^{2}$ = 0.118]. See **Figure 4** and **Table 1** for means and standard errors.

As in Experiment 2, the significant main effect of Test contrast showed that children performed better on the Easy contrast than the Difficult contrast. Across exposure types, children were more able to successfully discriminate the more different-sounding languages (Spanish vs. Mandarin) than the more similar-sounding languages (Spanish vs. Italian).

Our main question concerned the role of the unfamiliarity of the Exposure Language. We expected that the fact that the language was not used in the Test contrast alone – it was an incongruent exposure language – would not be enough for an exposure language to facilitate performance. Instead, we hypothesized that the Exposure Language also needed to be unfamiliar to facilitate the subsequent test discrimination. To test this prediction, we conducted a set of planned orthogonal comparisons designed to separate unfamiliarity and incongruence. In the first contrast, we compared the performance of children in the Congruent Language exposure conditions (e.g., exposed to Mandarin and tested on the Easy contrast in Experiment 1, and exposed to Italian and tested on the Difficult contrast in Experiment 2) to the performance of children in both the Incongruent-Unfamiliar and Incongruent-Familiar Language exposure conditions (e.g., exposed to Mandarin and tested on the Difficult contrast in Experiment 1, exposed to Italian and tested on the Easy contrast in Experiment 2, and exposed to English and tested on both contrasts in Experiment 3). That difference was not significant [*F*(1,114) = 0.61, *p* = 0.43], suggesting that lack of participation in the Test contrast alone did not facilitate discrimination.

In the second comparison, we compared children in the Incongruent-Unfamiliar Language conditions (exposed to Mandarin and tested on the Difficult contrast in Experiment 1, exposed to Italian and tested on the Easy contrast in Experiment 2) with children exposed to English, which was the Incongruent-Familiar Language (Easy and Difficult contrasts in Experiment 3). Here, the difference was significant [*F*(1,114) = 9.67, *p* = 0.002, $\eta_{p}^{2}$ = 0.078]. Children exposed to English in Experiment 3 performed significantly worse than those exposed to an Incongruent-Unfamiliar Language in Experiments 1 and 2. There were no interactions between these Exposure Language contrasts and the Test contrast. Thus, the unfamiliarity of the Exposure Language, not merely its relation to the test materials, affected performance across Test contrasts.

As in our prior studies, we were also interested in examining whether age played a role in children’s performance. There was a significant effect of age [*F*(1,118) = 12.28, *p* = 0.0006, $\eta_{p}^{2}$ = 0.094], suggesting that older children were more successful overall. We also included age (in months) in an interactive linear model, along with Exposure Language and Test contrast. The overall model was significant [*F*(11,108) = 4.877, *p* < 0.0001, $\eta_{p}^{2}$ = 0.332], and we also saw a significant interaction between age and Test contrast [*F*(1,108) = 4.80, *p* = 0.03, $\eta_{p}^{2}$ = 0.062], revealing that for older children, there was a greater difference between the Easy and Difficult contrasts than for younger children. For the Easy contrast, the simple effect of age was significant [*t*(108) = 4.44, *p* < 0.0001], while the simple effect of age was not significant for the Difficult contrast [*t*(108) = 1.26, *p* = 0.21]. This pattern shows that older children performed significantly better than younger children on the Easy contrast, but age did not affect performance on the Difficult contrast. There were no other significant interactions, suggesting that across ages, the Exposure Language affected children’s performance similarly.

### Discussion

In Experiment 3, we found that children who were exposed to English, the Incongruent-Familiar Language, performed significantly worse on the subsequent language discrimination task than children who were exposed to an Incongruent-Unfamiliar Language. As in Experiment 2, children performed better on the Easy contrast than on the Difficult contrast. From these results, we can conclude that in order for the Exposure Language to significantly facilitate discrimination, the language must be both incongruent with the Test contrast *and* unfamiliar.

Förster (2009) and Förster et al. (2009) have suggested that novelty can change how stimuli are processed. For example, participants may be asked to attend to either the global or local features of ambiguous Navon figures, and processing these features may draw on different attentional resources (Navon, 1977; Heinze et al., 1998). When the task is framed as novel, participants are then slower to make decisions about the local elements than those participants for whom the task is made to seem more familiar (Förster et al., 2009). The authors propose that this may be an adaptive strategy. Global processing may be more appropriate when faced with unknown stimuli, as the individual does not yet know what to expect and can then adjust more easily by focusing on broader properties. Similarly, global processing may encourage a focus on similarities, while local processing highlights differences (Förster, 2009). In our discrimination task, it could be that attending to more global properties, such as pitch variation or prosody, is more useful than attending to more local details, such as specific lexical items. Therefore, the relative novelty of the Incongruent language may actually help children focus on the more relevant cues that distinguish the two Test languages, rather than highlighting the irrelevant, local differences between the two utterances spoken by the target speaker.

## General Discussion

In three experiments, we sought to understand how language exposure changes children’s sensitivity to the differences between unfamiliar languages. In Experiment 1, we saw that without additional language exposure (Control condition), children found it easier to discriminate between typologically distinct languages (Mandarin and Spanish) than typologically similar languages (Italian and Spanish). Contrary to our initial predictions, hearing an unrelated language did not hinder children’s ability to make a difficult discrimination. Instead, we found that across test contrasts, exposure to an Incongruent Language (an exposure language that is not involved in the language discrimination itself) actually facilitated children’s ability to discriminate between two unfamiliar languages. Children who were tested on Spanish vs. Italian after hearing Mandarin (Experiment 1, Difficult contrast) or Spanish vs. Mandarin after hearing Italian (Experiment 2, Easy contrast) performed better than children who were tested on Spanish vs. Mandarin after hearing Mandarin (Experiment 1, Easy contrast) or Spanish vs. Italian after hearing Italian (Experiment 2, Difficult contrast). Additionally, we learned that this facilitation only occurs with unfamiliar languages; hearing a familiar language (English) does not improve children’s ability to discriminate between novel languages (Experiment 3).

Contrary to our expectations, exposure to an unfamiliar language did not enhance children’s ability to discriminate that particular language from another unknown language, unlike adult participants in a prior study by Bond et al. (1998). However, in that study, the exposure and test materials were produced by different speakers. Thus, one possible explanation for this unexpected result comes from research on speaker identification. Experience with an unfamiliar language improves adults’ ability to identify voices in that language (e.g., Thompson, 1987; Goggin et al., 1991; Köster and Schiller, 1997; Winters et al., 2008). Listeners can identify a bilingual speaker across both of the speaker’s languages, suggesting that adults are sensitive to indexical properties that are not language-specific (Winters et al., 2008; Wester, 2012). Increased experience with a language can also improve talker identification (Goggin et al., 1991; Winters et al., 2008; Bregman and Creel, 2012). Thus, experience with a language may encourage at least older listeners to pay attention to indexical properties and focus on those characteristics of an utterance.

If experience with a language helps listeners recognize individual voices, it may be that hearing the same language in both the exposure and test phases encouraged children to attend to indexical information, or local properties of the speaker’s voice. As discussed earlier, attention to local properties may also emphasize the differences between two stimuli, potentially making it harder for children to determine which utterance matched the target sentence (Förster, 2009). In contrast, the presence of an Incongruent exposure language may have highlighted cross-language dimensions that were ultimately more useful in this task. That is not to say that indexical features are irrelevant to the task; in our design, language and indexical information were confounded, and the task could have been solved entirely by using indexical cues. However, given that all our speakers were female and we controlled other properties such as volume, specific cues about the speaker may not have been as useful as the more global, cross-language differences. Therefore, children who heard a Congruent Language may have been limited by focusing primarily on the talker-specific cues.

Talker identity can be informative, and both infants and young children often consider it to be a potentially important source of information (e.g., Jerger et al., 1993; Houston and Jusczyk, 2000). Younger infants focus enough on speaker cues that they may have trouble generalizing across speakers of different genders, while older infants can succeed, demonstrating that infants must learn to ignore acoustic cues that initially salient (Houston and Jusczyk, 2000). Interestingly, infants may only be sensitive to talker identity in their own language; they do not notice a change in talker in a novel language, again demonstrating the intertwined relation between processing speaker information and processing language (Johnson et al., 2011). Children, too, have difficulty separating linguistic and indexical information. For example, preschool-aged children have difficulty separating meaning and speaker gender: 3- to 6-year-olds showed significant interference when they heard a male voice say “mommy” or a female voice say “daddy” (Jerger et al., 1988). Interference decreased with age, suggesting that children gradually get better at ignoring irrelevant indexical information and focusing on the critical aspect of the speech (in this case, meaning).

This possibility – that children gradually develop the ability to ignore irrelevant indexical information – is also consistent with the age effects found in Experiment 3. If younger children are performing the task by primarily relying on indexical cues, that would explain why they perform equally across the Easy and Difficult contrasts. By focusing on properties of the speaker’s voice, younger children may not be able to take advantage of the greater cross-language differences that exist between Spanish and Mandarin, compared to Spanish and Italian. Older children may have been better able to use these additional cues, leading them to be more successful on the Easy contrast.

The ability to focus on the appropriate cues for a given task is an important one, and research in a variety of domains reveals that contextual cues can affect what features are salient. For example, in a categorization task, Perry et al. (in preparation) found that having outliers in a category set changed participants’ perception of the similarity of objects in the set. In their task, adult participants were asked to arrange items such as keys such that more similar tokens were placed closer together. When the set included an outlier (e.g., an object that was significantly larger than the other objects), participants judged other items as more dissimilar, suggesting that having a highly distinct token warps the similarity space. This finding is consistent with earlier work by Goldstone (1994) revealing that categorization training can change what dimensions participants consider to be relevant. In our task, the presence of highly dissimilar Incongruent-Unfamiliar Language may have made the Test languages seem more dissimilar to each other and thus improved children’s ability to discriminate between them.

The highly dissimilar Incongruent-Unfamiliar Language also adds overall increased variability to what children are hearing, and increased variability has also been shown to help listeners determine which cues are likely to be important, even if the variability is in a task-irrelevant dimension (e.g., Nygaard and Pisoni, 1998; Maye et al., 2002; Apfelbaum and McMurray, 2011). For example, hearing labels from multiple speakers can make it easier for infants to learn similar-sounding words, perhaps by increasing the salience of the critical contrast and de-emphasizing other acoustic differences (Rost and McMurray, 2009). Adults, too, benefit from variable training when learning new phonetic categories, identifying unfamiliar accents, and understanding new speakers (e.g., Lively et al., 1993; Clopper and Pisoni, 2004; Bradlow and Bent, 2008). In our task, exposure to an Incongruent Language may have provided additional variability, allowing children to more readily ignore irrelevant features, such as the rhythm of a new language, and changed what features they considered when deciding which utterances matched. In contrast, neither the Congruent-Unfamiliar language nor English (Incongruent-Familiar) provided new information to guide children’s attention.

One limitation of the current design is that we have no way to know what properties of the stimuli children are using to make the test discrimination. Future studies could tease apart potential sources of information. For example, exposure to a multi-speaker passage might discourage children from attending to indexical properties and thus improve younger children’s performance. Alternatively, we could test children on a single language (e.g., Spanish vs. Spanish Test contrast), where they would need to use indexical information in order to identify the target speaker. In that case, we would predict that children should perform better after exposure to a Congruent language, which could highlight indexical information, while an Incongruent language would be less informative in drawing attention to less relevant cross-language features. A third possible method for better understanding the role of indexical and language cues would be to adjust our English-only training phase, which is used to familiarize children with the task. In the current method, children may be inadvertently encouraged to focus on indexical cues, given that they are taught to perform the task using a male vs. female speaker distinction. We could have instead drawn attention to language with a training phase that pitted an English speaker against a speaker of a novel language, for example. That, in turn, could make language more salient than talker and might also boost performance.

Ultimately, our goal is to better understand how children perceive new languages that they may encounter in the real world. Earlier, we reviewed evidence that children consider language to be an important social cue, preferring to affiliate with those speakers who sound the most familiar. In addition, children also use the way a speaker sounds to make inferences about the individual. For example, they think that a speaker with an unfamiliar accent is likely to wear less familiar clothing (Wagner et al., 2014a). However, both our data and prior studies have shown that children may have difficulty categorizing non-native speakers (e.g., Floccia et al., 2009). We do not know if children in our task recognized that monsters across trials spoke similarly, or if they expected those monsters to share traits. Future research could explore how children integrate their social and perceptual understanding of unfamiliar languages.

One way that we can explore this integration is by considering children who actually encounter multiple languages in their daily lives. Bilingual children, like their monolingual peers, prefer native-accent speakers, demonstrating that they, too, are biased by familiarity (Souza et al., 2013). However, regular exposure to multiple languages may also increase children’s sensitivity to the languages in their environment. Bilingual toddlers, for example, can appropriately determine which of their languages to use with an unfamiliar adult (Genesee et al., 1996). In addition, infants may not even need to have regular bilingual exposure for experience with different languages to affect their behavior. Even when they come from monolingual homes, infants growing up in more linguistically diverse neighborhoods are more likely to imitate a non-native speaker than infants in more homogenous environments, demonstrating how exposure to unfamiliar languages can change how young children perceive speakers of unknown languages (Howard et al., 2014). Together, these studies demonstrate that regular exposure to multiple languages may change how infants and children respond to the languages that surround them. While our study did not include children who were bilingual, future studies could explore whether brief exposure to new languages also influences the language discrimination abilities of children who already have experience hearing different languages.

What is intriguing in our findings is that even very brief experience with a new language was enough to change children’s perception of unfamiliar languages. Across studies, we have shown that hearing a novel language appears to change the information to which children attend when performing a discrimination task. We therefore suggest that exposure to multiple languages, in the absence of proficiency, may increase the salience of differences between languages and ultimately improve children’s ability to determine who can communicate with whom.

## Conflict of Interest Statement

The authors declare that the research was conducted in the absence of any commercial or financial relationships that could be construed as a potential conflict of interest.
