# Supplementary material for: The role of experience in children’s discrimination of unfamiliar languages
Source: Front Psychol. 2015 Oct 15;6:1587. doi: 10.3389/fpsyg.2015.01587 (PMC4606017; doi:10.3389/fpsyg.2015.01587)
Supplement: Supplementary file 1 [file Data_Sheet_1.DOCX]

**Appendix**

Sample Exposure Passages

***Mandarin (Study 1*)**

duo1chi1 se4la1 neng2 bu3chong1 ying2yang3.

xiang4pian1 mo2hu4 ying1gai1 chong2xin1 pai1.

da4jia1 zong3 shuo1 se4la1 yao4 chang2 chi1.

xiu1 mo2hu4 ren2xiang4 fang1fa3 hen3duo1.

ta1 ren4wei2 se4la1 bu2 gou4 jian4kang1.

gai4nian4 mo2hu4 ying1 qu4 zhao3 lao3shi1.

*Eating lots of salad is good for health.*

*Out-of-focus photos require retakes.*

*People always say one should eat salads more often.*

*There are many methods to fix an unclear portrait.*

*He thinks salads are not healthy enough.*

*If [one is] unclear of the concepts [he/she] should find the teacher.*

***Italian (Study 2)***

Corro in rapida fuga cercando le luci che si trovano in giardino

Le luci si trovano con le lumache vicino al melo senza luce

Il melo usa le lumache lucide di luglio

Quando in fuga mi piece riposarmi sotto il melo antico e lucido

Alcuni giorni ci si appendono lucchetti dal melo

La fuga verso la liberta e` anche lucrative

*I quickly run looking for the lights placed in the garden*

*The lights are located with snails near the apple tree without light*

*The apple tree uses lucid snails of July*

*While I'm escaping, I like resting under the old and lucid apple tree*

*Some days the locks are hung from the apple tree*

*The escape from freedom is also lucrative*

***English (Study 3)***

A strong wind blew the letter out of his hand.

One day Danny went to the museum.

Puppies love to meet new friends.

His favorite thing to do is to play with dolphin Jack.

They both had ice cream instead

Some people were waiting for the bus.

She mustn’t see it or the surprise will be spoiled.
